# Application of starter cultures to table olive fermentation: an overview on the experimental studies

**DOI:** 10.3389/fmicb.2012.00248

**Published:** 2012-07-19

**Authors:** Aldo Corsetti, Giorgia Perpetuini, Maria Schirone, Rosanna Tofalo, Giovanna Suzzi

**Affiliations:** Department of Food Science, University of Teramo,Mosciano Sant’Angelo, Teramo, Italy

**Keywords:** table olives, fermentation process, starter cultures, lactic acid bacteria, yeasts

## Abstract

Table olives are one of the oldest fermented foods and are considered as an important component of the Mediterranean diet, since their richness in monounsaturated fats (primarily oleic acid) and phenolic compounds may function as antioxidants in the human body; in the Western world they represent one of the most popular fermented vegetables but, despite its economic significance, table olive fermentation is still craft-based and empirical. In particular, such a type of fermentation results from the competitive activities among indigenous, contaminating microorganisms, the microbial balance depending on several intrinsic (pH, water activity, diffusion of nutrients from the drupe, and level of anti-microbial compounds) and extrinsic (temperature, oxygen availability, and salt concentration) factors. At present, to reduce the risk of spoilage and to achieve a more predictable process there is an increasing interest in developing starter cultures for table olives fermentation. Anyway, the application of starter cultures in the field of table olives is quite far from reaching the diffusion as it has in other sectors of food industry (e.g., dairy products and alcoholic beverages). This review focuses on experimental researches devoted to studying starter cultures for possible application to table olive fermentation both at artisan and industrial level.

## INTRODUCTION

The development of fermentation technologies is lost in the mists of history. Fermented foods and beverages such as bread, cheese, table olives, wine, and beer have been prepared and consumed for thousands of years and are strongly linked to culture and tradition, especially, in rural households, and village communities. Fermented foods can, in general, be described as palatable and wholesome foods prepared from raw or heated raw materials. The fundamental reasons for the development and acceptance of fermented foods can be variably ascribed to preservation, improved nutritional properties, better flavor/aroma, upgrading of substrates to higher value products, and improved health aspects. Microorganisms, by virtue of their metabolic activities, contribute to the development of peculiar properties such as taste, aroma, visual appearance, texture, shelf life, and safety (Hammes, 1990). At first, fermented foods were obtained through spontaneous fermentation but this kind of process often resulted in an uncontrollable fermentation, so different skills have been developed for controlling technical parameters during the fermentation processes. Moreover, experience has shown that back-slopping (i.e., inoculation of the raw material with a small quantity of a previously successfully fermented batch) accelerates the initial phase of fermentation and results in the promotion of desirable changes during the whole process. Actually in some sectors of food industry the fermentation is controlled through the inoculation of selected starter cultures.

## TABLE OLIVE FERMENTATION

Table olives are defined as pickled vegetables, that is those products, in which preparation and preservation is achieved by a combination of salting, fermentation, and/or acidification. The Trade Standard Applying to Table Olives of International Olive Oil Council (IOOC, 2004) defines table olives as “the sound fruit of varieties of the cultivated olive trees (*Olea europea L*.) that are chosen for their production of olive whose volume, shape, flash-to-stone ratio, fine flesh, taste, firmness, and ease of detachment from the stone make them particularly suitable for processing; treated to remove their bitterness and preserved by natural fermentation; or by heat treatment, with or without the addition of preservatives; packed with or without covering liquid.”

However, even if table olives are an important economic source for the producing countries the fermentation process is still empirical and far from being controlled.

In general, the fermentation is carried out by homo- and hetero-fermentative lactic acid bacteria (LAB) and/or yeasts and depends on the cultivar itself and on industrial and agricultural practices. The initial processing and the subsequent changes give rise to a microbial sequence that leads to ultimately to the dominance of those microorganisms and to the required product characteristics. Any deviations from the required environmental conditions can alter the microbial balance and result in a defective product. Under normal conditions the product in the bulk should have a pH around 3.8–4.2, a total titratable acidity 0.4–0.7% lactic acid, a residual lye of 0.09–0.11 N, and a salt concentration ranging from 4 to 8% (w/v) depending on various factors such as the final pH, addition of organic acids (e.g., acetic, lactic, citric), and temperature of storage (Montano et al., 2003). The main purposes of processing are the removal of fruit bitterness by hydrolysis of some phenolic compounds like oleuropein (Ciafardini et al., 1994), the achievement of a preservation effect, and the improvement of the organoleptic characteristics of the final product.

The two main commercial preparations of table olives obtained by fermentation process are:

### LYE-TREATED OLIVES

 By this method, even indicated as Spanish-style, bitterness is removed by adding lye (2.5–3% w/v). When the lye treatment finishes olives are washed with tap water in order to remove the alkaline solution. The washing should not be excessive because it could cause the lost of polyphenols and fermenting sugars. When the washing water is removed olives are placed in brines (10–11% w/v NaCl) in which they are maintained during the fermentation period that could vary from 3 to 7 months, after which olives are packaged in new acidified brine and sold.

Fermentation is driven by LAB, mainly belonging to lactobacilli and to a lesser extent to *Leuconostoc* and *Pediococcus* spp. (Flemming et al., 1985).

### DIRECTLY BRINED OLIVES

In this system, often referred as Greek-style, olives are put directly in brine with a salt concentration of about 6–10% (w/v; Balatsouras, 1990). In this process, the removing of bitter compounds is due to the enzymatic activities (mainly β-glucosidase and esterase) of indigenous microorganisms and to the diffusion of polyphenols to brine and it is slow and only partial (Garrido-Fernández et al., 1997; Tassou et al., 2002).

The fermentation process can last 8–12 months and it is mainly conducted by a mixed population of LAB and yeasts (Balatsouras, 1990; Kotzekidou, 1997; Brenes et al., 2004; Romero et al., 2004).

All these processes start spontaneously, so they are not fully predictable and are strongly influenced by the availability of fermentable substrates, salt content, pH, aerobic/anaerobic conditions and temperature control, as well as by the presence of microorganisms contaminating the drupe (Bobillo and Marshall, 1991, 1992; Garcia Garcia et al., 1992; Fernández González et al., 1993; Montano et al., 1993; Duran-Quintana et al., 1999; Spyropoulou et al., 2001; de Castro et al., 2002; Tassou et al., 2002; Alvarez et al., 2003; Chorianopoulos et al., 2005; Abriouel et al., 2011). To improve fermentation and produce consistent and high-quality final products, many authors recommend a strict process control of the above parameters besides the use of starter cultures, such as *Lactobacillus plantarum* (Etchells et al., 1966; Leal-Sánchez et al., 2003; Chorianopoulos et al., 2005; Lamzira et al., 2005; Marsilio et al., 2005; Sabatini et al., 2008), *L. pentosus* (de Castro et al., 2002; Panagou et al., 2003, 2008; Servili et al., 2006) or both (Sánchez et al., 2001; Panagou and Tassou, 2006).

## STARTER CULTURES

Starter cultures are defined as a preparation or material containing large numbers of variable microorganisms, which may be added to accelerate and improve a fermentation process (Holzapfel, 2002). Microorganisms selected to be used as starter cultures are expected to have some characteristics such as: adapting easily to the raw material and process, developing sensory quality, extending shelf life, reducing the processing time and energy during the production, inhibiting food-related pathogenic microorganisms, as well as having probiotic, non-pathogenic, and non-toxigenic properties. Moreover, the use of starter cultures allow the degradation of anti-nutritional factors, the improvement of protein digestibility, and bio-availability of micronutrients, and the nutritional enrichment of food through the biosynthesis of vitamins, essential amino acids, and other nitrogen compounds. Regarding shelf life, the use of starter cultures can enhance it by inhibiting spoilage microorganisms through the competition for nutrients and the production of inhibitors. In fact, it is well known that LAB used as starter cultures can produce a wide range of anti-microbial compounds such as organic acids, carbon dioxide, hydrogen peroxide, diacetyl, ethanol, bacteriocins, reuterin, and reutericyclin able to inhibit or reduce undesirable flora (Holzapfel, 2002). Since the food industry has to face with consumers’ demand for longer shelf life and safety of minimally processed foods and free from chemical preservatives, some authors proposed the use of bacteriocin-producing strains as starter culture for olive fermentation (Ruiz-Barba and Jimenez-Diaz, 1994; Leal-Sánchez et al., 2003; Delgado et al., 2005; Arroyo-López et al, 2005). In fact, the *in situ* production of bacteriocins may increase the competitiveness of the producer strain in the food matrix and contribute to the prevention of food spoilage leading to a more controlled and standardized product (Ross et al., 2002; Leroy and De Vuyst, 2004). Also yeasts can be used as biocontrol agents, in fact they are able to produce non-desirable yeast species inhibiting factors. Santos et al. (2000) demonstrated that *Pichia membranifaciens* produces a toxin, called CYC 1106, active against *Candida boidinii* in presence of a salt concentration ranging from 0.1 to 1 M. Also, another yeast species such as *Wicheramomyces anomalus* is able to release substances showing inhibitory activity against a wide range of microorganisms (Passoth et al., 2011). It is evident that their use should allow the reduction of salt and preservatives concentration and ensure packaging stability (Arroyo-López et al 2012).

Selected starter cultures are widely used in some sectors such as alcoholic beverages and dairy products but they are not very common in table olive fermentation. Vaughan et al. (1943) proposed for the first time the use of a starter culture for table olive fermentation. In particular, they recommended the use of starter culture for Manzanilla olives after observing the difficulty to ferment them by applying the classic Spanish method. Some years later, Borbolla y Alcalà et al (1952), after conducing different experiments on starter cultures, stated that the addition of pure culture of lactobacilli was important to ensure the normal evolution of fermentation and prevent spoilage. But it was only in the 1980s that the first commercial starter for table olive fermentation was prepared (Brighigna, 1984). The authors developed a procedure for preparing pure, spray-dried cultures of *L. plantarum*. This method guaranteed both an easy handling and a high survival rate when cells were revitalized in a medium made up of NaCl 8.5 g/l, glucose 10 g/l, yeast extract 3 g/l, and having pH 6.4. However, in spite of this good premises the use of starter cultures for table olive fermentation is still limited, probably due to different reasons, as a basic lack of knowledge on how to select, apply, and control the starter performances; the observation that in some cases spontaneous fermentation can be quite well controlled and a high-standardized product is not desired in all the markets; limitation of the biodiversity of the fermentation imposed by a commercial starter. Regarding this, the use of a starter can lead to the loss of authenticity of the product. Recent advances in the field of strain selection and application are expected to contribute to the development of new starters with increased diversity, stability, and industrial performances.

### SELECTION CRITERIA

The selection of starters is based on diverse criteria including homo-fermentative metabolism, high acidification rate and fast consumption of fermentable substrates, salt, organic acids and medium polyphenol tolerance, flavor development, temperature range for growth, oleuropein-splitting capability, and bacteriocin production (Duran-Quintana et al., 1999; Delgado et al., 2005; Hurtado et al., 2012). Another important characteristic of a starter culture is its ability to dominate the indigenous microbiota (Daeschel and Fleming, 1984; Daeschel et al., 1987). Dominance of the starter culture would be exerted by its fast and predominant growth under fermentation conditions and/or its ability to produce antagonistic substances (Marugg, 1991). In addition, for commercial purpose, it is necessary that starter cultures resist to freezing or freeze-drying process. Moreover, the pasteurization of raw material could be used to eliminate the competitive microorganisms and to make olives more fermentable. Chorianopoulos et al. (2005) successfully used a *L. plantarum* strain as starter in pasteurized olive fermentation and observed that the rate of pH drop was not as high as in unheated olives, but final pH values and acid development were more pronounced.

One of the latest research topic in this field is the use of fermented table olives as probiotic carrier. For that purpose, microorganisms should play a dual role both as starter and probiotic culture, which made it possible to control fermentation processes and achieve the final probiotic product with probiotic characteristics.

Starting from various table olive cultivars and fermentation processes, different microorganisms (**Table 1**) have been studied, indicating that the success of inoculation depends both on the cultivar and the processing method (Panagou and Tassou, 2006; Hurtado et al., 2010, 2012). In general, the final concentration of the inoculum ranges from 10^6^ to 10^7^ cfu/ml of brine. Only for *L. paracasei* an inoculum of 10^9^ cfu/ml was used.

**Table 1:** Main starter cultures used for table olive fermentation in experimental studies.

Starter	Cultivar	Reference
*L. plantarum*	Bella di Cerignola	Perricone et al. (2010)
	Manzanilla	Duran-Quintana et al. (1999)
	Hojiblanca	Ruiz-Barba et al. (2010)
	Conservolea	Chorianopoulos et al. (2005)
	Picholine	Lamzira et al. (2005)
	Manzanillo	Leal-Sánchez et al. (2003)
	Moresca and	Sabatini et al. (2008)
	Kalamata	
*L. pentosus*	Arbequina	Hurtado et al. (2010)
	Itrana and Leccino	Servili et al. (2006)
	Manzanilla	Sánchez et al. (2001)
	Gordal	Bautista Gallego et al. (2011)
*L. plantarum/*	Conservolea	Panagou et al. (2008)
*L. pentosus*		
*L. plantarum/*	Kalamon	Tsapatsaris and Kotzekidou (2004)
*D. hansenii*		
*L. pentosus/*	Green olives	Segovia Bravo et al. (2007)
*S. cerevisiae*		
*E. casseliflavus/*	Manzanillo	de Castro et al. (2002)
*L. pentosus*		
*L. paracasei*	Bella di Cerignola	De Bellis et al. (2010)

### APPLICATION OF STARTER CULTURES FOR TABLE OLIVE FERMENTATIONS

LAB play an important role during table olive fermentation, in fact they are able to enhance the olive preservation due to a progressive acidification of the fermenting brine with a consequent pH decrease and the production of antimicrobial substances and bacteriocins (Ruiz-Barba and Jimenez-Diaz, 1994; Marsilio et al., 2005). Moreover, they also improve the aroma and flavor characteristics of the product (Borcakli et al., 1993). So, a correct inoculum of selected strains allow to improve the product quality (Garrido-Fernández et al., 1997; Sánchez et al., 2001; de Castro et al., 2002; Leal-Sánchez et al., 2003). LAB mainly considered in studies dealing with starters selection are *L. plantarum* (Etchells et al., 1966; Leal-Sánchez et al., 2003; Chorianopoulos et al., 2005; Lamzira et al., 2005; Marsilio et al., 2005; Sabatini et al., 2008) and *L. pentosus *(de Castro et al., 2002; Panagou et al., 2003, 2008; Servili et al., 2006). All the above cited studies demonstrated that these microorganisms have the potential to improve the microbiological control of the process, increase the lactic acid yield, and provide the production of high quality fermented olives. In particular, Servili et al. (2006) selected a strain of *L. pentosus *(1MO) and used it as a starter to ferment, in pilot plant at controlled temperature of 28°C, black olives (Itrana and Leccino cv.) in brines modified for pH value (pH 6), carbohydrate content (0.3% w/v glucose), and growth factors (0.05% w/v yeast extract), obtaining olives debittering in 8 days. Some authors proposed the use of strains able to produce bacteriocins as starter cultures because this ability could facilitate their dominance over the natural microbial population. With this aim, *L. plantarum* LPCO10 has been successfully used as a starter culture in olive fermentation (Ruiz-Barba and Jimenez-Diaz, 1994; Leal-Sánchez et al., 2003). This strain was isolated from Spanish-style fermented green olives and it was able to produce two bacteriocins, namely plantaricins S and T (Jimenez-Diaz et al., 1993). These two bacteriocins were found to be active against a number of natural competitors of *L. plantarum* and also against bacteria that can cause olive spoilage (Ruiz-Barba et al., 1991). In particular Leal-Sánchez et al. (2003) compared the fermentation profile of green olives produced by a spontaneous fermentation vs. the inoculation with *L. plantarum* LPCO10. This microorganism was able both to dominate the fermentation process and to induce a rapid decrease of pH during the first phases of fermentation reducing olive spoilage. Moreover, it induced a higher free total acidity.

Recently, Panagou et al. (2008) evaluated the effect of a mixed starter culture on the fermentation of natural black olives (cv. Conservolea) made up of *L. plantarum* and a commercial preparation Vege-Start 10 (Chr. Hansen’s Biosystems, Horsholm, Denmark) based on pure freeze-dried *L. pentosus* appropriate for vegetable fermentation. Such a strain was evaluated in a fermentation trial in presence of a brine containing 6% (w/v) NaCl and a temperature of 20°C for an overall period of 30 days. In all the cases, during the period of fermentation the salt concentration was maintained constant at the initial level of 6% by periodical additions of coarse salt. Both starter cultures were effective in establishing an accelerated fermentation process and reducing the survival period of Gram-negative bacteria by 5 days compared with the spontaneous process, thus minimizing the likelihood of spoilage. Also the acidification of the brines was similar for the two selected starters, but *L. pentosus* showed a better performance than the *L. plantarum* strain, which may be less adapted to the olive fermentation conditions due to his different origin.

Similarly, Hurtado et al. (2010) found that *L. pentosus* showed better fermentation performances than *L. plantarum*; on the basis of their results, those authors stated that *L. plantarum* was not suitable for controlling *Arbequina* table olive fermentation. In this case, the fermentation was performed at 20°C for 52 days in a brine containing 8% NaCl (w/v). Once fermentation ended the brine was replaced with new sterile 5% NaCl (w/v) brine containing 1% acetic acid (v/v) and olives were stored at 4°C to stop any possible microbial development. The reported data indicate a clear effect of temperature on the starter culture activity; in fact, it can influence microbial metabolism and fermentation capacity. In general, for *L. plantarum* and *L. pentosus* the most appropriate temperature to obtain a good fermentation ranges from 20 to 25°C.

As mentioned before another important characteristic of starter cultures is their ability to grow at low temperature. This trait can become of great importance especially in winter when the low temperature could delay the microbial activity. Duran-Quintana et al. (1999) demonstrated that by using a selected strain of *L. plantarum* as starter it was possible to carry out a normal Spanish-style green olive fermentation at low temperature (12°C) by using 3% NaCl and correcting pH to 5.0 with HCl. Similar results were obtained by Sánchez et al. (2001), by using a selected strain of *L. pentosus *inoculated in lye-treated green olives at alkaline pH.

Recently, Lavermicocca et al. (2005) suggested the use of table olives to develop a probiotic food. Following a similar approach, De Bellis et al. (2010) proposed the use of the probiotic strain; *L. paracasei* IMPC2.1 as starter for olive fermentation. This bacterium was able to colonize the gut of healthy and constipated subjects. In general, the use of probiotic strains provide additional distinguished health and nutritional benefits to table olives: strong free-radical scavenging action and atherogenesis prevention (Visioli et al., 2002); delay of cellular aging due to vitamins A, B, and E and their precursors (Garrido-Fernández et al., 1997); increase of high-density lipoprotein cholesterol related to monounsaturated fatty acids (Lavermicocca et al., 2005). In this case, the process was carried out in different brining conditions (4 and 8% (w/v) NaCl), at room temperature and at 4°C, showing that the probiotic strain successfully colonized the olive surface dominating the natural LAB population and decreasing the pH of brines to ≤5.0 after 30 days until the end of fermentation. The obtained results showed that *L. paracasei* IMPC2.1 was also able to reduce the survival period of potential spoilage microorganisms.

Recent studies showed that the growth of LAB can be increased by the simultaneous inoculation of yeasts. In fact, yeasts can produce some substances such as vitamins (B_1_ and B_6_), amino acids, purines, or break down complex carbohydrates essential for the growth of *Lactobacillus* spp. (Abbas, 2006). Segovia Bravo et al. (2007) showed that *L. pentosus* performances during green olives fermentation are improved in presence of *Saccharomyces cerevisiae*. Similar results were obtained by Tsapatsaris and Kotzekidou (2004). In this case, when *Debaryomyces hansenii* was inoculated 48 h before *L. plantarum*, the growth rate of *L. plantarum* was increased probably due to the fact that some yeast strains are able to produce nicotinic and pantothenic acids, biotin and vitamin B_6_, which are essential for *L. plantarum* growth. Hurtado et al. (2010) showed that the co-inoculation of *L. pentosus* and *Candida diddensiae* for Arbequina table olives fermentation reduced *Enterobacteriaceae* survival, influenced yeast diversity during the first stage of fermentation, and improved the sensorial quality of olives. Moreover, an inhibition of both contaminating yeasts and food-borne pathogens and the improvement of LAB development were observed. Other yeasts known to be prevalent in fermented table olives such as *C. boidinii* or *P. membranifaciens* should be studied as possible co-starter yeasts (Arroyo-López et al., 2008).

Moreover, enterococci seem to be involved in table olive fermentation; in fact, different authors reported their presence in Spanish-style green olives (Floriano et al., 1998; Franz et al., 1999; de Castro et al., 2002; Ben Omar et al., 2004). Randazzo et al. (2004) isolated four strains of enterococci belonging to the species *Enterococcus faecium*, *Enterococcus casseliflavus*, and *Enterococcus hirae* from naturally fermented green olives collected from different areas of Sicily region (Italy). Some authors proposed the use of enterococci as starter culture for Spanish-style green olive fermentation. In particular, Lavermicocca et al. (1998) and Deiana et al. (1992) suggested the use of *E. faecium* associated with *L. plantarum* or *S. cerevisiae*, respectively, to improve olive fermentation.

Moreover, de Castro et al. (2002) suggested the use of *E. casseliflavus* as starter because of its VanC phenotype, which is an inherent (naturally occurring) low-level resistance to vancomycin. This property is not transferable, and is related to the presence in the chromosome of the *vanC-2* gene (Murray, 1998). *Enterococcus* *casseliflavus* was inoculated at the beginning of Spanish-style green olive fermentation (cv. Manzanilla), together with *L. pentosus*. This last was added at the same time as the enterococci or 24 or 48 h thereafter. In all cases there was a decrease of both fermentation time and growth of spoilage microorganisms. Sequential inoculation increased the survival of lactobacilli, in particular, their survival was higher when *L. pentosus* was inoculated 48 h after brining. This type of inoculation produced a faster brine acidification, a greater consumption of carbohydrates and a rapid pH decrease. Therefore, *E. casseliflavus *should be used as starter culture because it is able to tolerate high pH without the drawback of transmissible antibiotic resistance shown by other proposed species such as *E. faecium*, which could hinder their utilization.

## CONCLUSION

Undoubtedly, the potential use of starter cultures for olive fermentation is not yet well understood; in fact, even if table olives market is booming, olive production is still mainly based on the work of artisans, without the addition of microbial starter or the application of advanced techniques. The prospect of applying starter cultures become attractive for food industries, thanks to the reduction of costs (e.g., energy), fermentation times, risk of spoilage (increased shelf-life), to the improvement of process control, sensory quality, and safety attributes. Moreover, some strains (e.g. *L. pentosus* 1MO) has been already successfully used in pilot plant indicating the strong potential for their usage for olive fermentation during industrial olive production (Servili et al., 2006).

Exploiting the activities of LAB, yeasts, and enterococci in table olives requires fundamental knowledge about their ecology, physiology, biochemistry, and molecular biology. At present, this seems the most rationale approach, indispensable for developing strategies aiming at obtaining safe product showing a high standardized quality preserving, in the mean time, the biodiversity and genetic resources as a basis for further starter strains development.

## Conflict of Interest Statement

The authors declare that the research was conducted in the absence of any commercial or financial relationships that could be construed as a potential conflict of interest.
